# The Influence of Vibro-Assisted Abrasive Processing on the Surface Roughness and Sub-Surface Microstructure of Inconel 939 Specimen Made by LPBF

**DOI:** 10.3390/ma16237429

**Published:** 2023-11-29

**Authors:** Joanna Radziejewska, Michał Marczak, Piotr Maj, Dominik Głowacki

**Affiliations:** 1 Institute of Manufacturing Technology, Warsaw University of Technology, 85 Narbutta Street, 02-524 Warsaw, Poland; joanna.radziejewska@pw.edu.pl; 2 Faculty of Materials Science and Engineering, Warsaw University of Technology, Woloska 141 Street, 02-507 Warsaw, Poland; piotr.maj@pw.edu.pl; 3 Faculty of Power and Aeronautical Engineering, Warsaw University of Technology, 00-665 Warsaw, Poland; dominik.glowacki@pw.edu.pl

**Keywords:** drag finishing, IN939, additive manufacturing, abrasive finishing, vibratory surface processing, surface integrity

## Abstract

This paper presents the research results on the influence of vibration abrasive machining parameters on the surface layer integrity of elements made by LPBF technology from Inconel 939. The research was carried out on samples of various sizes on vibrating smoothing machines. The influence of the size of the processed elements, the type of abrasive shapes, the processing time, and the supporting fluid on the surface roughness and microstructure of the processed elements was analyzed. Tests have shown that as a result of using vibration processing, it is possible to reduce the surface roughness five times to the value of Ra = 1.1 µm. A significant influence of the type of abrasive shapes was found. There was no significant effect of the machining fluid on the process.

## 1. Introduction

Nickel (Ni) superalloys are in high demand for critical turbine components due to their exceptional mechanical strength at elevated temperatures and superior resistance to corrosion and oxidation [1,2]. As the aerospace industry focuses on reducing carbon emissions and improving fuel efficiency, the need for materials that can withstand even more extreme temperatures, resulting from higher engine temperatures, has intensified [3]. This ongoing quest drives the exploration of Ni superalloys capable of operating at even higher temperatures.

In this context, additive manufacturing (AM) is gaining popularity in the aerospace industry due to its numerous advantages, including weight reduction, the ability to produce complex shapes, reduction in the number of components, and cost savings [4]. Notably, Inconel 939, a nickel-based superalloy designed for high-temperature applications in gas turbines, stands out with its impressive operating temperature of up to 850 °C, remarkable corrosion resistance, and weldability. The demand for components made of Inconel 939 is surging, particularly in challenging environments.

Components manufactured from IN939, characterized by moderately weldable characteristics, are typically produced through investment casting [5]. The standard thermomechanical treatment for IN939 involves an initial solution treatment step, aimed at dissolving undesired phases that may form during solidification, including both the γ’ (Ni_3_(Al,Ti)) and the problematic η phase. Subsequently, a two-step aging treatment fosters the formation of a fine dispersion of strengthening γ′ and carbide particles [5]. Despite the alloy’s potential, there have been only limited studies in open literature exploring the processing of IN939 parts using methods like Laser Powder Bed Fusion (LPBF) [6,7,8,9] or electron beam Powder Bed Fusion (PBF) [10].

The microstructure of as-built IN939 parts, as reported in these studies, consists of columnar grains that are notably smaller than those found in components produced through conventional casting. These grains are predominantly oriented with a <001> crystalline direction parallel to the building direction (BD) [11]. Furthermore, the presence of an intragranular cell structure formed by elongated cells parallel to the BD is common [7]. The distribution of precipitates in as-built specimens remains a subject of debate, with conflicting evidence regarding the presence of γ′ precipitates, carbides, and the η phase [5,11]. Previous studies have underscored the challenges of producing LPBF-processed IN939 samples with minimal crack density and residual porosity [7]. During the printing process, the microstructure features lenticular pools oriented perpendicular to the building direction. This structure is susceptible to segregation and inclusions at melt-pool boundaries due to rapid heat transfer during the laser heating process, resulting in stress concentrations and intergranular cracking. These issues are common in AM nickel-based alloys and contribute to hot cracking, along with other defects like balling, lack of fusion, and keyhole formation [12].

A persistent issue in additive manufacturing (AM) is the high surface roughness observed in printed objects, often necessitating additional post-processing finishing procedures. These treatments can potentially alter the properties of the surface material layer, which can have an impact on mechanical characteristics and operational performance, particularly for intricate printed components. The most frequently used finishing methods involve machining processes like turning, milling, and drilling, which can achieve the desired accuracy and surface smoothness. However, these techniques encounter challenges when dealing with complex shapes, thin-walled parts, and materials that are difficult-to-machine, such as nickel-based superalloys and titanium alloys, which are commonly used in the aviation industry, but have poor workability [13,14].

One of the commonly utilized post-processing approaches for 3D printed parts involves abrasive processing using loose abrasive shapes. Recent studies [15] have utilized barrel finishing to machine elements made of Ti6Al4V and Inconel 718. These studies have revealed that the change in surface roughness depends on the initial morphology of the printed object and the accessibility of the surface to the abrasive medium. However, barrel finishing has the drawback of extended processing durations. In the case of aircraft blade profiles made from Inconel 718 using the selective laser melting (SLM) method, rotational drum machining has been employed to smooth and strengthen these components [16]. This process involves the use of steel shot for strengthening purposes. The results of this treatment have shown increased microhardness near the surface, the transformation of tensile stresses into compressive stresses, a reduction in surface roughness, and a decrease in near-surface porosity, in line with findings from prior research [17].

In this current study, the Inconel 939 alloy and the impact of abrasive finishing on post-AM parts’ heat treatment was analyzed. This study aims to evaluate the effects of heat treatment and abrasive machining on micro-components manufactured from Inconel 939 using LPBF technology. The evaluation will encompass changes in surface texture, material microstructure, and mechanical properties, with a particular focus on small parts where these changes can substantially affect performance. The study will investigate surface integrity after LPBF, manufacturer-recommended heat treatment in air and argon atmospheres, and abrasive machining performed on samples after AM and heat treatment.

## 2. Materials and Methods

### 2.1. Samples Preparation

The tests were carried out on samples made of Inconel 939 powder, the chemical composition of which is given in Table 1. Inconel 939 is a nickel-chromium alloy that balances high-temperature resistance, corrosion, and oxidation resistance. This material is difficult-to-machine due to the high content of alloying elements. The mechanical properties of the alloy after printing using the LPBF method and heat treatment, depending on the adopted printing strategy, are presented in Table 2.

**Figure 1:**
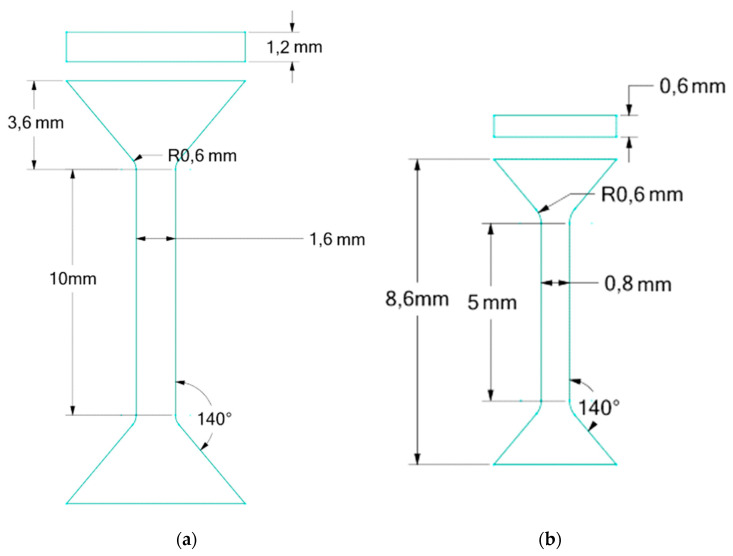
Shapes and dimensions of the specimens (green) produced by LPBF in Inconel 939 (**a**) Midi, (**b**) Mini.

Using the LPBF method, 2 types of small paddle-shaped samples with symmetrical ends were produced: Midi and Mini (Figure 1), with a thickness of 1.2 mm and 0.6 mm. They were produced using an EOS M 100 3D printer (EOS GmbH, Krailling, Germany). Powder with a grain size of 15–40 μm was used for printing.

The printer settings included

- a rotation between layers of 67 degrees,
- a layer thickness of 20 µm,
- the hatch distance was 50 µm,
- a 5 mm width for scanning stripes with 0.1 mm overlap,
- building direction is along the height of the sample (a laser exposure strategy developed by EOS to improve the quality of the material) [20].

**Figure 2:**
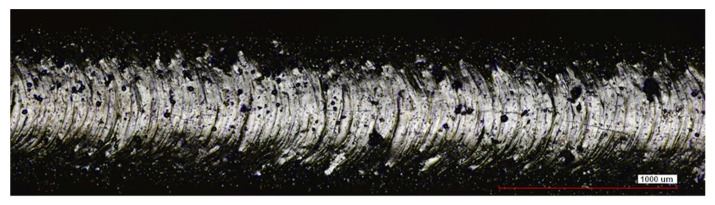
Surface of an Inconel 939 sample after LPBF viewed in a confocal microscope.

Various laser power setups were tested, with E (volumetric energy density) ranging from 58 J/mm^3^ to 91 J/mm^3^. The sample (Figure 2) printed with E = 75 J/mm^3^, the parameter used in the article, exhibited the best material properties in terms of density and hardness.

These samples were heat treated in accordance with the manufacturer’s documentation (BIBUS HOLDING AG, Fehraltorf, Switzerland) [18]. Heat treatment was carried out in three stages: saturation annealing at 1374 K for 4 h and rapid cooling in air, then annealing for 6 h at 1000 °C and annealing at 800 °C/4 h in stir argon.

The specimens depicted in Figure 1 were vertically aligned with their height in accordance with the printing direction during the manufacturing process. These samples were arranged in rows, ensuring an approximate spacing of 1 mm between each specimen.

### 2.2. Experimental Station and Measurements

Vibration treatment of elements after LPBF printing and heat treatment was carried out on a specially designed and built station for small elements. The station consisted of 6 work chambers. After preliminary tests, it was determined that the fastest mass loss occurs when vibrations with an amplitude of 1.4 mm and a frequency of 30 Hz are used. Each pair of Midi and Mini samples with different dimensions was assigned a specific type of abrasive shapes (Table 3) and processed for 4 h. The selected abrasive shapes differed in size, weight, and the material from which they were made. The aim of the research was to determine the influence of machining conditions, i.e., the type of abrasive tools, the influence of supporting fluid and machining time on the material removal rate and surface roughness.

Each pair of Midi and Mini samples were subjected to vibration abrasive processing with the additional use of ROLLKEMIK FE-L 120-B32 machining fluid (Rollwasch Italiana S.p.A., Albiate, Italy) and without any fluid. The liquid used is intended for finishing and polishing materials, and additionally contains antioxidants. The pH value is 8.4. The manufacturer recommends it for use with ceramic, porcelain, and metal fittings.

The samples were cleaned in an ultrasonic bath for 3 min after each hour. Subsequently, the weight loss was checked using a WPS 50/C/2 scale with an accuracy of 0.5 × 10^−5^ g (Radwag, Radom, Poland). The last stage was to measure the roughness of each sample three times using a Taylor Hobson profilometer (Taylor Hobson, Leicester, England), from which the average value of the roughness parameters was calculated. The length of the measurement section for Midi samples was 12 mm with cut-off λs = 2.5 mm, for Mini samples, the measurement section was 4 mm and λc = 0.8 mm due to the small dimensions of the sample. The surface roughness parameters such as: Ra, Rq, Rt, Rp, Rv, Rz, Rsk, Rku, RSm, RS were analyzed for samples after printing and after 4 hours of abrasive treatment. The course of roughness changes after each hour was analyzed based on the Ra and Rz parameters. 

Microstructure analyses were performed on cross sections made perpendicular to the building direction in the middle of the testing parts of samples. Samples were grinded and polished on diamond grain suspension with sizes of 9, 3, and 1 micrometer.

The research was conducted on a confocal laser microscope (Keyence VK-X100) and SEM Joel 7000. For selected samples, chemical analysis Energy Dispersive X-ray Spectroscopy (EDS) was performed on a cross-section.

## 3. Results and Discussion

### 3.1. Vibratory Surface Processing with Fluid

Table 4 shows the weight loss in grams for samples after vibration processing using six different abrasive shapes. Despite the low weight of the samples in relation to the weight of the abrasive shapes, there was no phenomenon of samples “floating” on the surface of the workpiece.

Analyzing the above mass loss graph for the Midi samples, it can be seen that the largest mass loss occurred for the Midi 1 samples processed with cubic and Midi 6 abrasive shapes, where ceramic pellets were used, while the smallest for the Midi samples 3, 4 and 5. In all cases, the process was most intensive in the first hour of processing.

Analyzing the mass changes for the Mini samples, the largest mass loss occurred for the Mini 1 sample where cubic abrasive shapes were used, Mini 5—resin cones, and Mini 6—ceramic pellets. In comparison, the most negligible mass loss was found for the Mini 4 sample (ceramic rods). Regardless of the size of the processed samples, the process was most intensive for the abrasive shapes with the largest mass.

Based on the results of preliminary tests, the abrasives ceramic cubes and ceramic pellets (the first and the last abrasives in Table 4) were selected for further finishing.

For selected abrasive shapes, the smoothing process was carried out again, lasting four hours. Each container with a given type of abrasive shapes contained eight Midi samples and eight Mini samples, which were weighed before processing. The entire process took place in the same conditions as the previously tested elements. Finally, all samples were cleaned in an ultrasonic bath for 3 min and then weighed. The results are presented in Table 5 below. The greatest weight loss occurred for ceramic cube-type abrasive shapes, both for Midi and Mini elements. The process was clearly more intense for the smaller Mini samples, and a 2.6% relative weight loss was found after 4 h of processing than for the Midi samples (1.6%). This is related to a more favorable ratio of the mass of samples to the mass of abrasive shapes in the case of Mini samples.

Surface roughness tests were carried out before and during abrasive treatment. Each sample was measured on a profilometer in three different places of the working part along the sample axis, and then the roughness parameters were determined. The measured data were averaged and are presented in Table 6 and Table 7. The measurements of the samples after printing showed high values of the roughness parameters Ra = 6.62 µm, Rz = 41.1 µm. The high surface roughness is due to the presence of unmelted powder particles strongly bonded to the surface, visible in Figure 2. This nature of the surface results in a large dispersion of the roughness parameter values in particular the maximum heights of picks and valleys Rp, Rv and the parameters Rt and Rz. Surface roughness analysis did not show any significant differences in surface texture between Midi and Mini samples (Table 6).

As a result of abrasive treatment, powder particles were removed, but in the initial stage of the process, it could be observed that the process did not proceed with the same intensity, more particles were removed at the edges of the samples than in the central area, which was the reason for significant differences in roughness parameters depending on the measurement location. As a result of abrasive treatment, the height of irregularities was reduced by 60–80% (Table 7).

The decrease in roughness depends on the type of abrasive shapes used. The most significant reduction in the roughness height was found for samples Midi 2, 5, and 6 and Mini 2, 4, and 6. Analyzing the standard deviation of the Ra parameter (Table 8), the most significant changes were found for samples Midi 1 and 6, which is consistent with the measured weight losses of the samples. Roughness intensively decreases in the first hour of the process regardless of the abrasive shapes used, while the last hour introduces slight changes in the surface texture (Table 9).

Even faster removal of roughness peaks can be observed in the range of Rz parameters (Table 9). The most intense decrease in the maximum roughness heights Rz can be observed in the first hour of processing for both Mini and Midi samples. As a result of four-hour processing, the Rz parameter values were several times lower, reducing them from 41.5 μm after printing to 7.3 μm for abrasive processing using ceramic pellets.

In the first hour of processing, the reduction in mass and roughness height (Ra, Rz) of the samples results mainly from the removal of large particles that are less strongly bonded to the substrate. This happens as a result of samples colliding with abrasive shapes. The process is most effective for ceramic, cubic and pellet shapes with the highest mass. After this stage, in the subsequent hours of processing, material loss occurs as a result of the micro-cutting process of the tops of surface irregularities, which is less intense due to the high hardness of the processed material. At this stage, it is more effective to use ceramic cubic shapes with sharp edges. The type of shapes used has a stronger impact on the material loss than on the relative changes in the roughness height (Ra), which range from 61% for roller-shaped abrasive material to 73% for ceramic pellets (Midi samples). For smaller samples, the efficiency of the process is higher than for larger elements. This is related to the greater weight share of particles on the surface in relation to the weight of the samples than for Midi samples. For both Mini and Midi samples, the differences in the surface roughness obtained after four hours of the process are relatively small. Observations using a confocal microscope did not show any deformations of the processed elements despite their small thickness (0.6 mm for Mini samples) and the large weight of the abrasive elements.

Table 10 presents a complete analysis of the surface texture for Midi and Mini samples after four hours of abrasive treatment using ceramic pellet. The nature of changes in the geometric structure of the surface is similar for both analyzed cases. The parameters describing the heights of inequality Ra, Rz, Rt, Rv, Rp decreased several times. The greatest changes can be observed in the parameter Rp describing the height of picks. This is related to the removal of powder particles from the surface. Also, the slopes of irregularities after machining are much smaller (Rda). However, smaller changes can be found for the horizontal parameters Sm and RS, which depend on the LPBF printing conditions. Surface roughness is significantly lower for Mini samples, which is related to the previously mentioned more favorable processing conditions.

### 3.2. Vibratory Surface Processing without Fluid

Abrasive machining tests were also carried out for a variant without machining fluid for ceramic cubes and ceramic pellets. As a result of processing, a relative change in mass was obtained for Midi samples of 2.3% and 2.2% for Mini samples in the variant using ceramic cubes (Table 11), which indicates more intensive processing than in the case of using liquid. The obtained surface roughness heights are smaller than for the machining variant using fluid (Table 12), Ra is 1.19 μm for Midi samples and 1.06 μm for Mini samples.

In the vibratory-abrasive process, adding the fluid increases the weight of the abrasive shapes and workpieces, generating more impact force while increasing the cutting force. However, the fluid reduces the coefficient of friction between the abrasive shapes and workpieces, resulting in reduced process efficiency. The number of effective impacts generating micro-cutting is reduced in favor of sliding the abrasive shapes on the surface of the workpieces. Hence, the decrease in roughness is more intense for dry machining.

**Figure 3:**
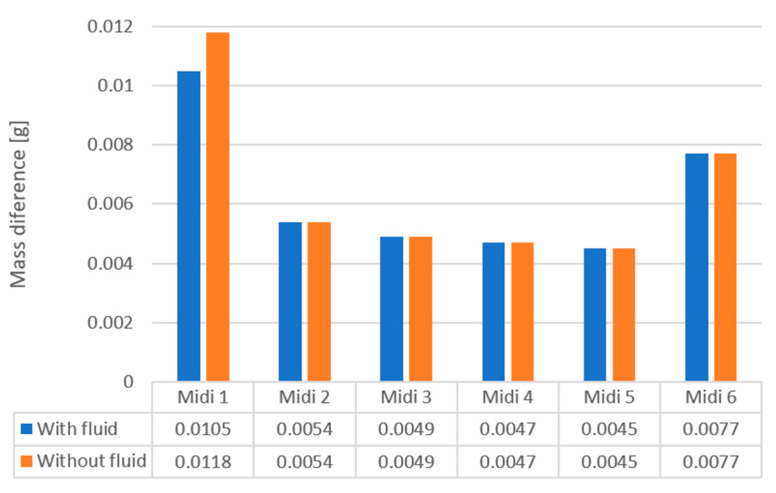
Comparison of the intensity of finishing machining for various variants of abrasive shapes with and without the use of machining fluid.

By comparing the intensity of vibration machining for both machining variants with and without the use of machining fluid, it can be concluded that there is no impact of the presence of machining fluid on the effects of abrasive machining. Only in the case of the first variant, where cubic shapes are used, the intensity of the process is clearly higher for processing without fluid. This is because this variant uses high-mass shapes with sharp edges, so the micro-cutting process is more intense without a supporting fluid. Figure 3 shows the test results for both processing variants.

**Figure 4:**
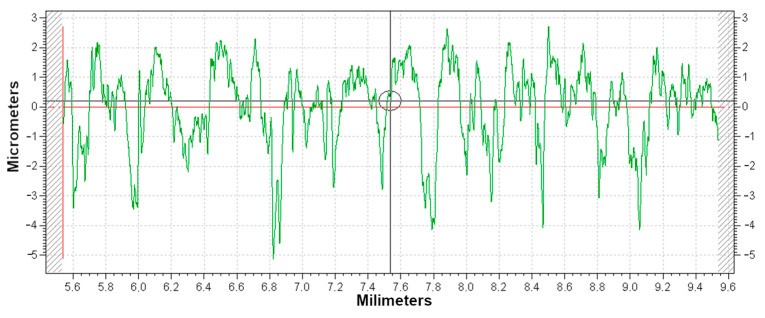
Surface roughness profile (green) of Midi 6 (Table 12) after 4 h of treatment without supporting fluid with ceramic pellets (Ra = 1.069 µm, Rz = 6.556 µm. Rq = 1.354 µm, RSm = 147.72 µm).

**Figure 5:**
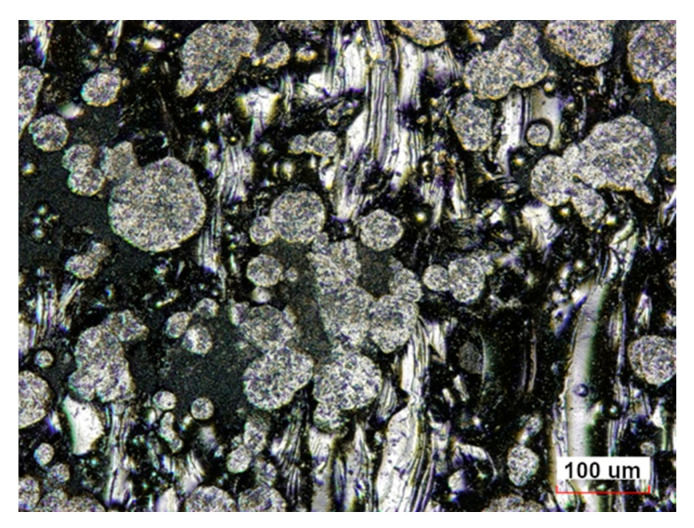
Surface of Midi sample after 4 h of treatment without supporting fluid with ceramic pellets (confocal microscope).

Figure 4 shows the surface roughness profile for the most favorable variant of vibration machining, i.e., Midi 6 in Table 12, after 4 h of machining without a supporting fluid with ceramic pellets. It is possible to observe decreased heights of the surface roughness peaks, which are clearly smaller than the roughness valleys. The photo of this surface (Figure 5) shows areas of picks from the traces of the micro-cutting process and valleys where this process did not take place.

### 3.3. Analysis of Microstructure and Microhardness

**Figure 6:**
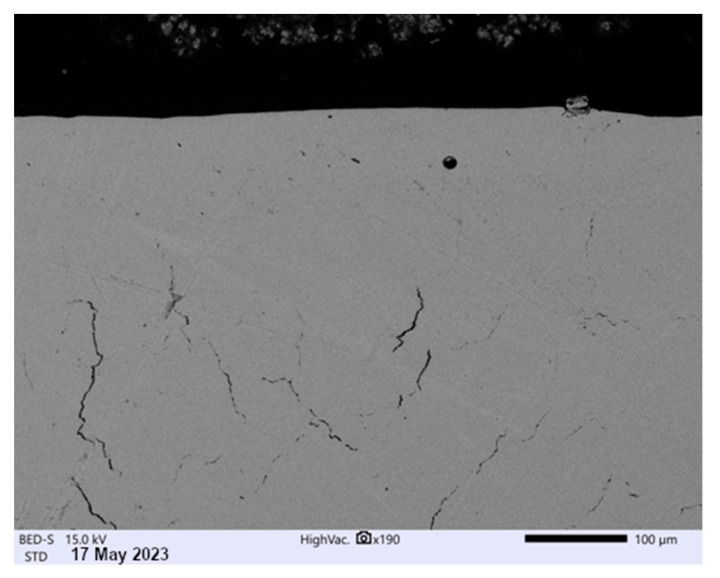
Microstructure of Inconel after printing LPBF (SEM).

Microstructure tests made on cross-sections have shown that all analyzed samples are free from defects typical for materials after printing, such as pores or cracks situated in the sub-surface layer. However, after heat treatment, a grid of microcracks was found in the central part of both small and larger samples, as seen in Figure 6.

**Figure 7:**
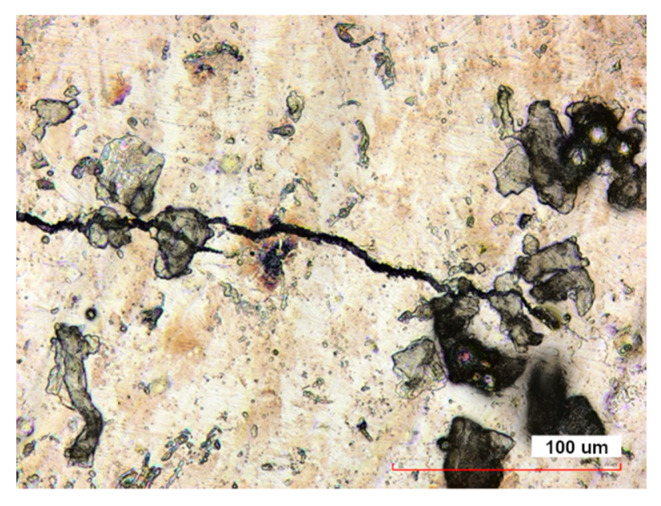
The surface of the sample Mini 1 directly after LPBF viewed in a confocal microscope displays visible microcracks.

Also, for smaller samples, the presence of single, shallow microcracks was found on the surface along the axis of the printed samples (Figure 7). Confocal and electron microscopy tests did not reveal the presence of this type of defect in the case of samples after heat and abrasive treatment. Due to the existence of a thicker oxide layer, confocal and electron microscopy did not reveal the presence of this type of defect in the samples after heat and abrasive treatments. The lack of surface microcracks in the case of Midi samples is related to different cooling conditions of the material after printing.

**Figure 8:**
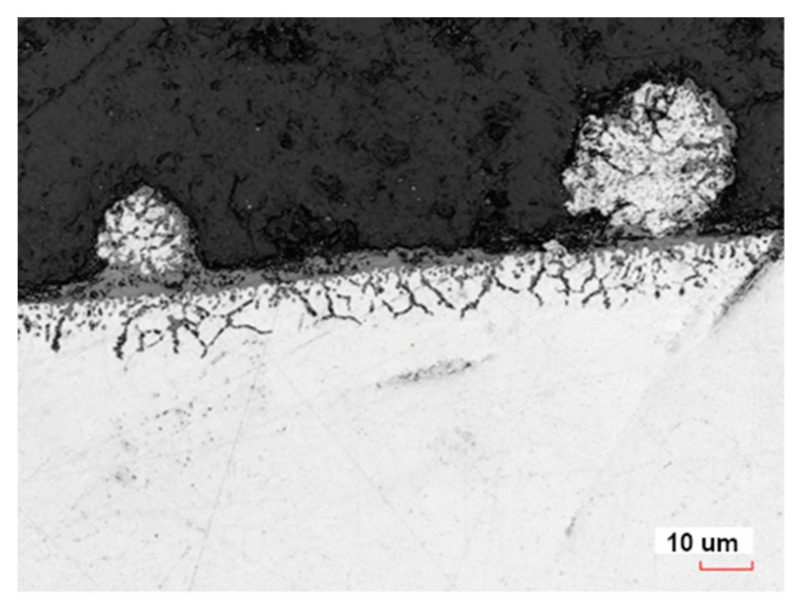
Microstructure of sub-surface after heat treatment.

For all tested samples after heat treatment, a continuous layer of oxides with a thickness of approximately several micrometers was found on the surface. Oxides were also found in the sub-surface layer with a thickness of approximately 10–15 µm (Figure 8), as well as in powder particles. After abrasive treatment, the thickness of this layer increased to 20–30 µm. It is likely that during abrasive treatment, oxygen diffused into the material and formed oxides.

**Figure 9:**
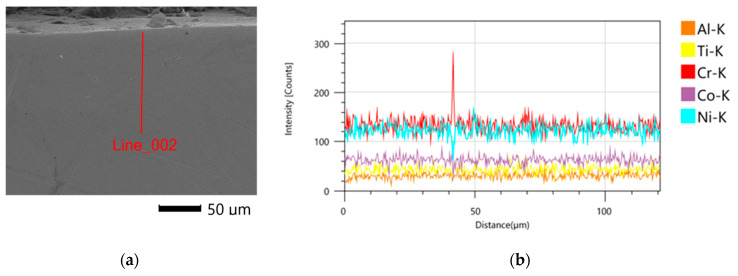
Midi sample after LPBF (**a**) location of chemical analysis (**b**) distribution of alloying elements in the sub-surface zone.

A linear analysis of the chemical composition of samples after printing and for samples subjected to heat and abrasive treatment was performed. The tests were carried out on metallographic cross sections perpendicular to the surface. The location of the analyzed area in relation to the sample surface is shown in Figure 9a. The Figure 9b shows exemplary results of the analysis of the distribution of alloying elements in the near-surface zone. It was found that the concentration of elements is constant in the tested zone for both Midi and Mini samples.

**Figure 10:**
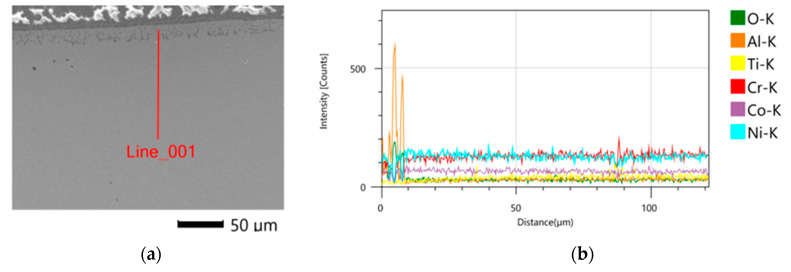
Midi sample after heat and abrasive treatment: (**a**) location of chemical analysis, (**b**) distribution of alloying elements in the sub-surface zone.

**Figure 11:**
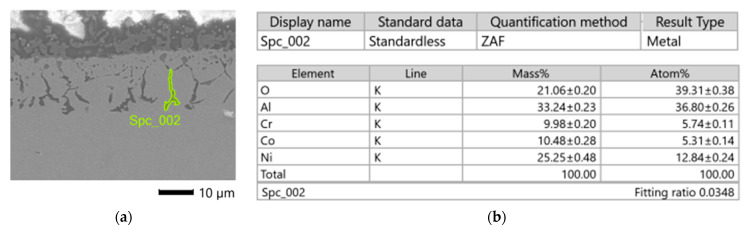
Sub-surface zone of the sample after heat and abrasive treatment: (**a**) location of the analyzed precipitation (**b**) chemical composition of the precipitation.

The chemical composition of the sub-surface layer changed as a result of heat and abrasive treatment. The linear distribution of elements is shown in Figure 10. In this case, the presence of oxygen and a higher concentration of Al in relation to the sample core were found. This is related to the presence of precipitation observed in this zone (Figure 11). In a zone with a thickness of about 20 μm, oxygen was found, which was not present in the case of printed samples. Reduced chromium concentrations in the near-surface zone can also be observed. The distribution of the remaining analyzed elements is homogeneous.

Chemical composition tests carried out for five precipitates in different areas of the sub-surface zone showed that their chemical composition is relatively constant. Table 13 shows results of analysis.

Microhardness tests were carried out on cross-sections for samples after LPBF printing, heat treatment, and heat and abrasive treatment. The lowest hardness was found for the sample after printing, approximately 500 HV. In the sub-surface zone with a thickness of approximately 50 μm, the microhardness was lower, 300–40 HV, and increased with the distance from the sample surface.

Similarly, in the case of the sample after heat and abrasive treatment, lower material hardness was found in the near-surface zone than in the core. Heat treatment resulted in a significant increase in the hardness of the material, in the core, the hardness was 680 HV, and in the near-surface zone where oxide precipitations were observed 400–500 HV. Abrasive processing did not cause changes in the hardness of the core material, but in the zone near the surface with a thickness of about 20 μm, an increase in hardness was observed compared to the heat-treated material by about 50 HV. This effect is probably related to plastic deformation caused by vibration processing.

### 3.4. Discussion

The primary objective of the present research was to enhance the surface integrity of Inconel 939, a nickel-based alloy, while concurrently reducing surface roughness. In pursuit of this goal, a series of tests were conducted to assess the condition of the surface. Notably, the findings underscore the pivotal role played by heat treatment in influencing the chemical composition, owing to diffusion-related phenomena [7]. An observed reduction in the concentrations of key elements, particularly aluminum and titanium, responsible for the formation of the strengthening γ’ phase, indicates that heat treatment significantly impacts the alloy’s composition [21]. While this reduction aids the smoothing process, it poses challenges to the material’s mechanical properties, leading to a compromise in strength [21,22].

In the subsequent phase, the analysis focused on the smoothed surface, employing various abrasive geometries. The obtained results revealed distinct roughness profiles, and a comparative assessment with similar studies highlighted noteworthy outcomes. Further discussion is warranted to delve into the implications of individual abrasive shapes on the smoothing process. Of the six tested variants of abrasive shapes used to smooth the surface of small elements, the most favorable effects were achieved for cubic and pellet ceramic abrasive shapes. These shapes were characterized by a larger mass of 3.8 g for ceramic cubes, and 1 g for ceramic pellets compared to rollers and resin cones weighing in the range of 0.31–0.75 g. This caused the energy of collisions with the processed elements to be higher and the process of removing particles from the material surface to be faster. Abrasive machining with the aid of fluid was slightly less effective than without it. This is related to the amortization of the energy of collisions between shapes and elements and the reduction of the friction coefficient in the presence of the supporting fluid. The material removal rate analyzed in the study showed that machining is most effective in the first hour of the process, regardless of the abrasive shapes used, the supporting fluid and the size of the processed samples. This is related to the removal of large particles that are less strongly bonded to the substrate from the surface. In the following hours, the process rate decreases because the micro-cutting process dominates. The relative loss of material is clearly greater in the case of smaller samples, this is related to the greater share of large particles on the surface in relation to the mass of samples than for larger samples. The obtained surface roughness after processing is similar, and a slightly more favorable surface texture of small elements is caused by the use of a shorter measurement section when measuring roughness.

Finally, the investigation extended to microstructure analysis, revealing a typical 3D printed element configuration characterized by melt-pools and elongated grains. Post-heat treatment observations unveiled the segregation of individual elements, accompanied by the occurrence of hot cracking—an issue inherent in 3D printed nickel-based alloys [23,24]. These findings contribute valuable insights into the challenges associated with post-processing steps and emphasize the need for strategies to mitigate hot cracking in 3D printed nickel-based alloys.

## 4. Conclusions

The research conducted to investigate the impact of vibro-abrasive finishing process parameters on material surface roughness and microstructure revealed that smooth surfaces can be achieved for small specimens fabricated through the LPBF method using Inconel 939. The research findings indicate that:

- Employing ceramic pellet shapes and machining fluid significantly reduces the average surface roughness Ra, from 6 µm to 1.2 µm.
- The type of abrasive shapes, especially their weight, exerts the most significant influence on the efficiency and surface roughness of the vibratory finishing process.
- It was observed that the impact of cutting fluid presence on machining results is dependent on the abrasive shapes used for ceramic and porcelain rollers. Better process efficiency was achieved without fluid.
- After heat treatment, a continuous layer of oxides several micrometers thick was found on the surface. The presence of oxides was also found in particles on the surface and precipitates in the subsurface layer.
- Microhardness tests demonstrated that following heat treatment, the material’s hardness increases from 500 HV0.01 after LPBF to 680 HV. The presence of sub-surface zones with a different microstructure and lower hardness compared to the core was found for elements after printing, heat treatment and vibration treatment.
- As a result of the diffusion of titanium and aluminum (the main components of the strengthening basic gamma phase) into the precipitates, a reduction in sub-surface hardness was found in the near-surface layer.
- In the sub-surface zone, the occurrence of oxide precipitates rich in aluminum was revealed for Midi and Mini elements after heat and abrasive treatment.
- A favorable balance between hardness and surface roughness was successfully attained in the process, highlighting its potential for utilization in advanced finishing methods for aerospace applications.

Further research is necessary to assess the impact of abrasive processing on the mechanical properties of small elements in which the share of the sub-surface layer, characterized by changes in microstructure and microhardness, is significant.

## Figures and Tables

**Table 1:** Chemical composition of Inconel 939 [18].

Element	Cr	Co	Ti	Al	C	Nb	Ta	W	Ni
Chemical composition [%]	22	19	4	2	0.2	1	1.4	1.9	Balance

**Table 2:** Mechanical properties of Inconel 939 printed by LPBF method [18].

	Heat Treated Condition		As-Manufactured	
	Vertical	Horizontal	Vertical	Horizontal
Yield strength Rp0.2 [MPa]	1100	1130	740	880
Ultimate tensile strength Rm [MPa]	1500	1520	1090	1160
Elongation	13	11	28	24
Hardness at room temperature ISO 6508 [19]	48		33	
Average density [g/cm^3^]	8.15			

**Table 3:** Description of the shapes used with sample descriptions.

Used for Samples	Abrasives			
	Type	Photo	Size [mm]	Mass [g]
Midi 1 Mini 1	Ceramic cubes	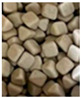	Irregular	3.8
Midi 2 Mini 2	Ceramic rods	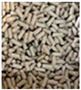	⌀4.1 × 14.5	0.5
Midi 3 Mini 3	Porcelain rods EB 0410 VZ	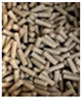	⌀9.5	0.37
Midi 4 Mini 4	Ceramic rods CB 0410 VH	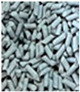	⌀4.1 × 11.5	0.31
Midi 5 Mini 5	Resin cones	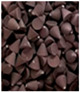	⌀11 × 11	0.75
Midi 6 Mini 6	Ceramic pellets	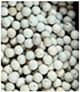	⌀6	1.0

**Table 4:** Weights of Midi and Mini samples during vibration processing using various abrasive shapes and the relative percentage weight loss after four hours of the process.

Sample	Time [h]					Relative Difference [%]
	0	1	2	3	4	
Midi 1	0.5206	0.517	0.5158	0.5139	0.5129	1.5
Midi 2	0.5181	0.5167	0.5153	0.5134	0.5127	1.0
Midi 3	0.5178	0.5154	0.5146	0.5139	0.5129	0.9
Midi 4	0.5182	0.5155	0.5155	0.514	0.5135	0.9
Midi 5	0.5161	0.5147	0.5136	0.5118	0.5116	0.9
Midi 6	0.5109	0.5073	0.5062	0.5044	0.5032	1.5
Mini 1	0.0607	0.0595	0.0594	0.0594	0.0594	2.1
Mini 2	0.0615	0.0609	0.0609	0.0605	0.0604	1.8
Mini 3	0.0620	0.0613	0.0612	0.0611	0.0610	1.6
Mini 4	0.0600	0.0594	0.0595	0.0590	0.0591	1.5
Mini 5	0.0617	0.0613	0.0611	0.0608	0.0603	2.3
Mini 6	0.0616	0.0610	0.0603	0.0602	0.0598	2.9

**Table 5:** Average results from mass measurements for Midi and Mini samples with fluid.

Sample	Cubes			Ceramic Pellets		
	Before	After	Relative Difference [%]	Before	After	Relative Difference [%]
Midi	0.5129	0.5045	1.6	0.5140	0.5087	1.0
Mini	0.06145	0.05982	2.6	0.06147	0.06055	1.5

**Table 6:** Surface roughness parameters for LPBF Midi and Mini samples.

Sample	Midi		Mini	
Roughness Parameters	Mean Value	SD	Mean Value	SD
Ra [µm]	6.62	0.90	6.38	0.24
Rsk	0.95	0.19	0.57	0.27
Rp [µm]	27.01	1.83	14.42	6.13
Rda [°]	17.74	0.96	9.35	0.24
RS [µm]	87.42	1.47	44.45	9.19
Rq [µm]	8.33	1.01	4.67	0.58
Rku	3.52	0.48	2.00	1.11
Rv [µm]	14.10	2.63	8.36	1.67
Rt [µm]	46.25	3.85	25.05	6.45
Rz [µm]	41.11	4.32	22.71	6.45
RSm [µm]	215.98	14.26	115.12	6.74

**Table 7:** Changes in the roughness parameter Ra in subsequent hours of abrasive machining of Midi and Mini samples and the relative change in Ra after four hours of machining.

Sample	Time [h]					Relative Difference [%]
	0	1	2	3	4	
Midi 1	6.623	3.061	2.773	2.412	2.342	64.6
Midi 2	6.623	4.832	2.650	2.216	2.018	69.5
Midi 3	6.623	3.544	3.035	2.665	2.598	60.8
Midi 4	6.623	3.690	2.842	2.846	2.486	62.5
Midi 5	6.623	2.847	2.804	2.167	2.238	66.2
Midi 6	6.623	2.086	1.739	1.958	1.811	72.7
Mini 1	6.380	1.457	1.595	1.375	1.563	75.5
Mini 2	6.623	2.726	1.582	2.147	1.150	82.0
Mini 3	6.623	1.146	2.892	2.373	2.273	64.4
Mini 4	6.623	2.880	1.029	1.362	1.042	83.7
Mini 5	6.623	3.374	3.162	0.965	2.721	57.4
Mini 6	6.623	1.534	1.713	1.624	1.209	81.1

**Table 8:** Changes in the standard deviation of Ra in subsequent hours of abrasive machining of Midi and Mini samples and the relative change after four hours of machining.

Sample	Time [h]					Relative Difference [%]
	0	1	2	3	4	
Midi 1	0.904	0.266	0.136	0.188	0.125	86.2
Midi 2	0.904	0.466	0.159	0.078	0.173	80.9
Midi 3	0.904	0.171	0.318	0.211	0.137	84.8
Midi 4	0.904	0.171	0.070	0.338	0.637	29.5
Midi 5	0.904	0.107	0.257	0.185	0.242	73.3
Midi 6	0.904	0.216	0.193	0.195	0.120	86.8
Mini 1	0.241	0.061	0.243	0.137	0.229	4.9
Mini 2	0.241	0.194	0.200	0.205	0.044	81.9
Mini 3	0.241	0.257	0.643	0.148	0.102	57.5
Mini 4	0.241	0.300	0.137	0.255	0.254	−5.3
Mini 5	0.241	0.221	0.124	0.093	0.264	−9.2
Mini 6	0.241	0.053	0.096	0.229	0.076	68.4

**Table 9:** Changing in roughness parameter Rz during abrasive treatment with fluid.

Sample	Time [h]					Relative Difference [%]
	0	1	2	3	4	
Midi 1	41.111	18.576	16.412	14.182	13.718	66.6
Midi 2	41.111	32.465	17.404	13.168	12.642	69.2
Midi 3	41.111	22.299	17.889	15.400	15.016	63.5
Midi 4	41.111	22.153	17.689	16.836	15.526	62.2
Midi 5	41.111	21.466	17.609	13.809	14.604	64.5
Midi 6	41.111	12.168	10.634	12.170	11.460	72.1
Mini 1	41.458	7.978	7.610	7.443	8.442	79.5
Mini 2	41.458	15.078	7.725	10.858	6.506	84.2
Mini 3	41.458	6.677	17.286	12.310	11.185	72.8
Mini 4	41.458	15.136	6.548	7.657	6.326	84.6
Mini 5	41.458	19.792	17.190	5.872	13.797	66.4
Mini 6	41.458	8.743	8.951	9.118	7.298	82.2

**Table 10:** Surface roughness parameters for Midi and Mini samples after four hour vibratory process using ceramic pellets.

Sample	Midi 6		Mini 6	
Roughness Parameters	Mean Value	SD	Mean Value	SD
Ra [µm]	1.81	0.12	1.21	0.08
Rsk	−0.16	0.15	−0.41	0.06
Rp [µm]	5.21	0.32	3.18	0.23
Rda [µm]	5.21	0.16	5.96	0.34
RS [µm]	68.75	1.01	35.05	3.73
Rq [µm]	2.23	0.11	1.54	0.10
Rku	2.78	0.40	3.07	0.34
Rv [µm]	6.25	0.76	4.11	0.52
Rt [µm]	13.56	0.71	8.62	1.20
Rz [µm]	11.46	0.60	7.30	0.73
RSm [µm]	237.14	12.95	171.50	13.71

**Table 11:** Average results from mass [g] measurements for Midi samples without fluid after abrasive treatment.

Sample	Time [h]					Relative Difference [%]
	0	1	2	3	4	
Midi 1	0.5177	0.5114	0.5111	0.5081	0.5059	2.30
Midi 2	0.5181	0.5167	0.5153	0.5134	0.5127	1.04
Midi 3	0.5178	0.5154	0.5146	0.5139	0.5129	0.95
Midi 4	0.5182	0.5155	0.5155	0.5140	0.5135	0.91
Midi 5	0.5161	0.5147	0.5136	0.5118	0.5116	0.87
Midi 6	0.5109	0.5073	0.5062	0.5044	0.5032	1.51

**Table 12:** Average results from roughness Ra [µm] measurements for Midi samples without fluid after abrasive treatment.

Sample	Time [h]					Relative Difference [%]
	0	1	2	3	4	
Midi 1	6.62	4.21	2.02	1.7	1.19	80.0
Midi 2	6.62	2.88	1.83	1.65	1.64	71.1
Midi 3	6.62	3.74	1.79	1.72	1.87	67.0
Midi 4	6.62	3.02	2.48	2.22	1.92	66.1
Midi 5	6.62	5.63	3.65	3.81	3.18	47.3
Midi 6	6.62	4.13	1.71	1.34	1.07	85.4

**Table 13:** Chemical composition of precipitates in sub-surface zone.

No	1	2	3	4	5
O	16.6	21.1	19.6	21.2	19.9
Al	29.6	33.2	31.1	35.7	31.4
Cr	11.2	9.9	10.4	9.4	10.6
Co	12.5	10.5	11.5	9.9	11.1
Ni	30.2	25.3	27.4	23.6	27.1

## Data Availability

Data are contained within the article.
